# Living on the Edge: Assessing the Extinction Risk of Critically Endangered Bonelli’s Eagle in Italy

**DOI:** 10.1371/journal.pone.0037862

**Published:** 2012-05-25

**Authors:** Pascual López-López, Maurizio Sarà, Massimiliano Di Vittorio

**Affiliations:** 1 Vertebrates Zoology Research Group, University of Alicante, Alicante, Spain; 2 Department of Environmental Biology and Biodiversity, University of Palermo, Palermo, Italy; Ohio State University, United States of America

## Abstract

**Background:**

The population of Bonelli’s eagle (*Aquila fasciata*) has declined drastically throughout its European range due to habitat degradation and unnatural elevated mortality. There are less than 1500 breeding pairs accounted for in Europe, and the species is currently catalogued as Critically Endangered in Italy, where the 22 territories of Sicily, represent nearly 95% of the entire Italian population. However, despite national and European conservation concerns, the species currently lacks a specific conservation plan, and no previous attempts to estimate the risk of extinction have been made.

**Methodology/Principal Findings:**

We incorporated the most updated demographic information available to assess the extinction risk of endangered Bonelli’s eagle in Italy through a Population Viability Analysis. Using perturbation analyses (sensitivity and elasticity), and a combination of demographic data obtained from an assortment of independent methods, we evaluated which demographic parameters have more influence on the population’s fate. We also simulated different scenarios to explore the effects of possible management actions. Our results showed that under the current conditions, Bonelli’s eagle is expected to become extinct in Italy in less than 50 years. Stand-alone juvenile mortality was the most critical demographic parameter with the strongest influence on population persistence with respect to other demographic parameters. Measures aimed at either decreasing juvenile mortality, adult mortality or decreasing both juvenile and adult mortality resulted in equivalent net positive effects on population persistence (population growth rate λ>1). In contrast, changes aimed at increasing breeding success had limited positive effects on demographic trends.

**Conclusions/Significance:**

Our PVA provides essential information to direct the decision-making process and exposes gaps in our previous knowledge. To ensure the long-term persistence of the species in Italy, measures are urgently needed to decrease both adult mortality due to poaching and juvenile mortality due to nest plundering, the top ranking mortality causes.

## Introduction

Estimating the risk of extinction of threatened species is a crucial aspect of population ecology and conservation biology [1]. A growing quantity of papers on this topic have been published in the last two decades, highlighting the importance of making quantitative predictions on population persistence [2]–[5]. For this purpose, population ecologists have used an assortment of analytical and simulation tools, enhancing our understanding about which demographic parameters are more influential on population’s fate. One of the most popular tools is the Population Viability Analysis (PVA) [2], [6]–[8]. Although PVAs were initially designed to estimate the likelihood of a population’s extinction [2], due to their flexibility, PVAs have been used in risk-assessment studies aimed at determining which demographic parameters are the most influential in population persistence [7], [9], [10]. Specifically, PVAs, by means of perturbation analysis, are able to examine the response of a model to changes in vital parameters, thus allowing the comparison of alternative management options [9], [11]–[14]. In addition, PVAs are able to incorporate spatial structure and environmental and demographic stochasticity into population models [15], [16]. Higher extinction risks are associated with species occupying high trophic levels, exhibiting a long-lifespan, delayed maturity, and breeding at a low population density in a small geographical range [3]. Such is the case with Bonelli’s eagle, *Aquila fasciata*, a threatened species for which population models have played an important role in informing management decisions aimed at eagle conservation [17]–[19].

Bonelli’s eagle is a large-sized raptor distributed across the Palearctic, Indo-Malayan and, marginally, the Afro-tropical regions [20]. The western Palaearctic population is irregularly distributed across the circum-Mediterranean area, encompassing the countries of northern Africa (Morocco, Algeria and Libya) and southern Europe (Portugal, Spain, France and southern Italy) [20]. In the second half of the 20th century, the species declined drastically throughout its European range owing to habitat degradation and unnatural elevated mortality, mainly due to direct persecution by hunters, electrocution on electric pylons and collisions with power lines [21]. Currently, the population appears to have stabilised, although the situation varies widely among regions, and the population’s size is far from being sufficient to ensure the survival of the species across Europe [22]. The species also faces local threats such as poisoning and poaching, persecution by pigeon fanciers, loss of prey species and increased human pressure on breeding habitats [23], [24]. As a consequence, the species has been listed as endangered in Europe [21], where less than 1500 pairs still breed, and 80% of these breeding pairs are in Spain. The species is listed on Annex I of the EU Wild Birds Directive (http://ec.europa.eu/environment/nature/legislation/birdsdirective/index_en.htm), and Appendix II of the Bern Convention (http://www.coe.int/t/dg4/cultureheritage/nature/bern/default_en.asp), as well as on the Bonn Convention (http://www.cms.int/documents/convtxt/cms_convtxt.htm) and CITES Convention (http://www.cites.org).

In Italy, historically, Bonelli’s eagle was recorded on the main islands (Sardinia and Sicily) and sporadically in the southern Apennines [25]. In Sardinia, the species was fairly abundant [26], but the population began decreasing in the 1960 s, and only 3–4 pairs remained in the late 1970 s [27]. Currently, certain proofs of presence are lacking (Schenk *pers. comm.*). In Sicily, Bonelli’s eagle breeding pairs were regularly recorded since the 19th century [28], [29]. In the 1960 s, the species disappeared from south-eastern Sicily due to heavy poaching. In the mid-1980 s, surveys recorded 17 breeding pairs [30]. Currently, the species breeds regularly in only 22 breeding territories [31], [32], representing nearly 95% of the entire Italian population (25–28 estimated pairs, authors’ unpub. data). As a consequence, the species is currently catalogued as Critically Endangered in Italy [33].

Given the delicate conservation status of Bonelli’s eagle in Italy, we incorporated the most updated demographic information to assess the extinction risk of the species in Sicily by means of a PVA. The specific objectives of this study were to i) estimate the risk of extinction under current conditions; ii) determine which demographic parameters have more influence on population dynamics using sensitivity analysis; and iii) simulate different population models through an elasticity analysis to explore the effects of possible management actions on the persistence of the species in Sicily.

## Materials and Methods

### Study Area and Data Collection

Sicily is located in southern Italy (from 38°18’N to 36°38’N and from 12°25’E to 15°39’E) and is the largest Mediterranean island, covering 25414 km^2^ (altitudinal range = 0 – 3322 m above sea level). Climatologically, it belongs to the Mediterranean region, with an annual rainfall ranging from 400 to 600 mm on the plains and from 1200 to 1400 mm in the mountains. Almost 24.4% of the territory is mountainous, 61.4% of the territory is highlands, and 14.2% of the territory is lowland. The natural vegetation has been reduced greatly by millennial human influence, and consequently, forests and Mediterranean vegetation account for less than 10% of the territory, which is located almost exclusively on the north-eastern ridge of the island. Habitat heterogeneity is evident in areas where cultivated zones (especially arable land) intermingle with artificial forest patches of *Pinus* and *Eucaliptus* spp., and in natural woodlands of *Quercus* spp. and Mediterranean vegetation. The island is one of the most populated areas in the western Mediterranean (195 inhabitants per km^2^).

#### Field procedure

We monitored Bonelli’s eagles from 1990 to 2010 as a part of an intensive field survey [30]–[32], [34]. Every year, all known territories and the surrounding potential habitats were surveyed by remote observation using terrestrial telescopes and binoculars to assess the desertion of sites and the detection of new pairs. Territory occupancy and the age of individuals (juvenile, immature, subadult and adult) were recorded, assuring at least three visits per site during each breeding season. Breeding parameters were assessed following the standard methodology for raptor monitoring [35]–[37]. Breeding success was calculated as the quotient between successful breeding pairs and pairs initiating reproduction [35]. Adult mortality rates were obtained indirectly from the juvenile recruitment rate. The juvenile recruitment rate was obtained through the estimation of turnover rates among adult pairs and mixed pairs (adult-subadult or subadult-subadult) [31], [37]. No statistically significant trends in the demographic parameters were detected, so we ruled out the existence of any sampling effort and/or annual effect on our results. No ringing was involved in this study. According to the Italian legislation, permission for observational and field studies are not necessary; hence, permits were not requested.

### Data Input and Model Construction

A Population Viability Analysis for the Sicilian population of Bonelli’s eagle was built using Vortex simulation software (version 9.93; http://www.vortex9.org). Vortex is an individual-based simulation software specifically recommended for PVAs [38], [39]. In brief, Vortex builds prospective stochastic age-structured population models, simulating a population by stepping through a series of events that describe the typical life cycle of sexual organisms: partner selection, reproduction, growth, mortality, emigration and immigration. Vortex was initially designed to study mammals and birds, such as Bonelli’s eagle, and is particularly useful for modelling the typical life of sexually reproducing, diploid organisms characterised by low fecundity rates, a long lifespan, local population sizes of less than 500 individuals, estimable age-specific fecundity and survival rates, and monogamous breeding [38]–[40]. The technical specifications of Vortex are fully detailed in [40].

In this study, the parameters of the life table were obtained by a combination of data from the published literature and intensive field sampling (see details about the different sources of data in Table 1). Once compiled, the parameters were introduced into Vortex to create baseline models based on current conditions (Table 1). Vortex was then used to compute both the intrinsic deterministic population growth rate (*det-r*) by classical analysis of the matrix population models [40], [41] as well as the intrinsic stochastic population growth rate (*stoc-r*) [40]. Once these values (*r*) were obtained, the population growth rate (λ) was calculated as λ = e*^r^* [41]. As recommended, all simulations were performed for over 100 years in 500 different iterations [42].

**Table 1:** Parameters used to construct individual-based models for the Population Viability Analysis (PVA) of Bonelli’s eagle in Sicily (Italy).

Parameter	Value	References
Number of runs (simulations)	500	10,17
Number of years for projection	100	17
Definition of extinction	just when one sex remains	17
Number of populations	1 (isolated population; i.e. immigration = emigration)	
Dispersal	not modelled	
Reproductive system	monogamous	20
Age of first offspring (both sexes)	3 years	20
Maximum age of reproduction	35 years	20
Maximum number of progeny per year	2 chicks	17
Sex ratio at birth (% males)	50%	17
Density dependent effects on reproduction	not modelled	
Mean and SD of females breeding (%)	Mean and SD of the % of successful pairs according to fieldwork inSicily for the period 1990–2010 (breeding success mean 60.15; SD 21.05)	present study
Number offspring per female per year (% ineach class)	Mean of the % of nests with 1 or 2 chicks according to fieldwork inSicily for the period 1990–2010 (50% 1 chick; 50% 2 chicks)	present study
Males in breeding pool (%)	100%	17
Specified age distribution	10 individuals of age (1); 8 of age (2); 42 of age (3); all the breedingpopulation in age (3); equal number of malesand females.	present study
Carrying capacity (K)	500 individuals	17
Harvesting	not modelled	
Supplementation	not modelled	
Mortality rates (in percentage):		
from age 0 to 1:	50.0^a^/52.1^b^	17,31/45
from age 1 to 2:	71.0^a^/43.0^b^	17,31/45
from age 2 to 3:	10.2^a^/43.0^b^	17,31/45
after age 3:	10.2^a^/13.0^b^	17,31/45

a Juvenile mortality from [17] based on satellite telemetry data. Adult mortality from [31] and fieldwork based on turnover rates.

b Juvenile and adult mortality from [45] based on capture-mark-recapture methods.

In models when individuals from only one sex remained alive, we considered the population to be virtually extinct [17], [43]. The probability of extinction was calculated as the proportion of iterations that were performed before a population became extinct after 100 simulated years. The precise age-class distribution in the population was not available for the species. Therefore, following the recommendation of [40], the initial population size was modelled as a stable age distribution (see [10] for a similar approach). The reproductive system was considered to be monogamous [20]. The Sicilian population of Bonelli’s eagle was modelled as a single isolated population. Although the eagles are highly mobile, especially juvenile birds [44], very few observations of birds crossing the Strait of Messina have been recorded. Hence, we ruled out the existence of a flux of individuals in the models (i.e., immigration and emigration rates were assumed to be equal).

Previous demographic analyses of Bonelli’s eagle populations suggested that mortality, both in adults [18], [19] and in juveniles [17], was the main vital rate regulating population size [45]. Unfortunately, current estimates of juvenile mortality for the Sicilian population of eagles were not available in the literature or according to fieldwork. Therefore, simulations were conducted using recent demographic information obtained from intensively surveyed populations (mainly in Spain and France). In our case, simulations were run under two different baseline scenarios: (i) the combination of juvenile mortality values recorded by satellite-tracking studies in eastern Spain [17] and adult mortality values obtained through the estimation of territorial turnover rates in Sicily [31]; and (ii) utilising both juvenile and adult mortality values obtained by means of systematic capture-mark-recaptures (CMR) in southern France [45] (Table 1). The use of independent sources avoided the potential biases that occur when using a single source of vital rates or with demographic data obtained by different methods for evaluating survival rates [46]. For simplification, the first baseline scenario was named “Spain” and the latter was named “France”.

Because the population size was low in comparison to regions of comparable dimension [32], we did not include density-dependent effects on reproduction in the models [43], [47]. In addition, following [17], [43], [48], the potential effects of inbreeding depression, catastrophes, harvesting, supplementation and genetic management were not included in the simulations [40]. Catastrophic events are unpredictable by nature and cannot be forecast; therefore, we decided not to include excessive uncertainty in the models. Neither harvesting nor supplementation are a concern in the species, and the simulation of inbreeding depression or genetic management was beyond the scope of this paper.

### Perturbation Analyses

Demographic perturbation analyses are a useful tool to explore how population growth rate (λ) responds to changes in vital rates (survival, growth and reproduction). Perturbation analyses included both sensitivity and elasticity analyses [12], [14]. The first analysis models change in “absolute terms”, i.e., varying a given parameter (e.g., adult mortality) by 0.01, 0.05, 0.10 and so on and then analysing how much that parameter affects the population growth rate (λ). In contrast, elasticity is a measure of the “proportional”’ effect of similar changes on different demographic parameters (i.e., the effect of similar changes at a fixed percentage: 1%, 2.5%, 5%, etc.) and their final effect on λ. Therefore, management strategies that simulate changes in demographic parameters with the highest impact on λ could be interpreted as being more important from a conservation point of view [9; but see 49].

#### Sensitivity analysis

In our case, to determine what demographic parameters have more influence on population trends, we simulated different scenarios including a range of values for i) adult mortality, ii) juvenile mortality, iii) breeding success, and iv) sex ratio at birth. These simulated scenarios were then compared to the baseline models (i.e., “Spain” and “France”). The simulations were performed varying one parameter at a time at fixed intervals while keeping the remainder of the parameters unchanged. In the case of sensitivity of adult mortality on λ, we modelled an increase from 0% to 25% at 2.5% intervals. Similar analyses were performed separately for both juvenile mortality and breeding success. The sensitivity of juvenile mortality on λ was modelled from 50% to 100% at 5% intervals. Breeding success was modelled from 0% to 100% at 10% intervals.

Bonelli’s eagle shows a high degree of reversed sexual dimorphism (i.e., females are on average 14% – 22% bigger than males) [20]. Because stochastic variation of sex ratio has long been considered a potential factor driving small populations to extinction [43], we also simulated changes in the sex ratio at birth (measured as the percentage of males) and measured their effects on the population growth rate and PE. To this end, the sex ratio at birth was modelled from 0% to 100% at 10% intervals.

The sensitivities were evaluated by the change in population growth rate (λ) resulting from a given change in demographic parameters as follows: *S_i_* = (λ*_i_* – λ*_b_)/*λ*_b_* x 100, where *S_i_* is the sensitivity of the model being investigated, λ*_i_* is the population growth rate of the model *i*, and λ*_b_* is the population growth rate of the baseline model. Calculated in this manner, sensitivity provides an indication of both the magnitude and the direction (positive or negative) of the change in λ. When the *S_i_* index is <0, the change causes the population growth rate to decrease; when the *S_i_* index is >0, the population growth rate increases.

#### Elasticity analysis

We simulated different models to explore the effects of possible management actions on the persistence of the species in Sicily. To this end, we again used the Spain and France baseline models, and then we calculated the effects on the population growth rate (λ) with a proportional increase or decrease of 5%, 10%, 15%, 20%, 25% and 30% in i) adult mortality, (ii) juvenile mortality, (iii) juvenile and adult mortality considered together, and (iv) breeding success. Changes in the sex ratio at birth were not modelled because it is not possible to manage the sex ratio. The parameters were modified one at a time. All other parameters of the model were kept unchanged. In all cases, we simulated scenarios based on reasonable options of population management following [17] and taking into account biological limits [49].

In both cases, the evaluation of the effects of changes in sensitivity and elasticity analyses was assessed on two demographic parameters: i) the probability of extinction (PE) in 100 years and ii) the annual rate of population growth (λ). Values of λ greater than 1 indicate that the population would increase, whereas λ values below 1 indicate that the population would decrease. Values of λ equal to 1 indicate that the population would remain stable.

## Results

### Baseline Models

Given the current conditions, population models indicate that the Bonelli’s eagle population in Sicily will decrease in the near future when taking into account the combination of demographic parameters from Spain and Italy (det-r *_Spain_* = −0.059) as well as those from France (det-r *_France_* = −0.067). Similar results were obtained in relation to the stochastic growth rate (stoc-r *_Spain_* = −0.064; stoc-r *_France_* = −0.074). In both cases, the population growth rate (λ) was below 1, indicating a population decrease (λ *_Spain_* = 0.938; λ *_France_* = 0.929). The models indicate that the Bonelli’s eagle population in Sicily would become extinct in less than 50 years (median time of extinction *_Spain_* = 44 years; median time of extinction *_France_* = 38 years; *N* = 500 simulations; Table S1). Considering both scenarios, Vortex forecasted a 100% probability of extinction in the next 100 years. Although these results should be taken cautiously (see the Discussion), they clearly show that the predicted population trend is negative given the current conditions.

### Sensitivity Analysis

**Figure 1:**
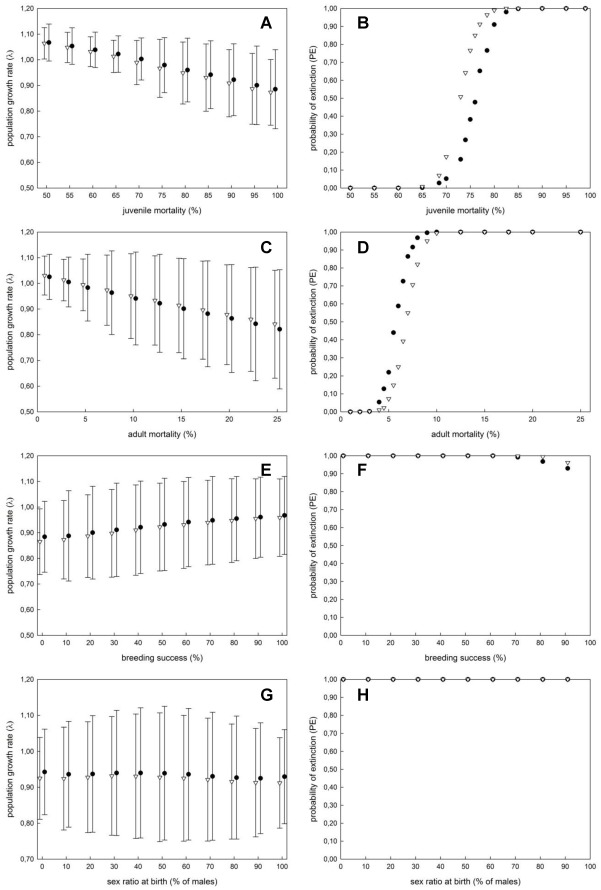
Sensitivity analysis of the population growth rate (λ) and the probability of extinction (PE) of the Bonelli’s eagle’s population in Sicily in relation to different values of juvenile mortality (a-b), adult mortality (c-d), breeding success (e-f) and sex ratio at birth (f-g). Simulations were run under two scenarios: (i) one including the combination of juvenile mortality values as reported in [17] based on satellite telemetry data, and adult mortality based on turnover rates in the Sicilian population [31] (black dots); and (ii) a second scenario using juvenile and adult mortality values as reported in [45] based on CMR methods (white triangles) (See text for further details). Note the sinusoidal shape of the curves of the PE in relation to juvenile and adult mortality, indicating that small variations in these demographic parameters may result in different values of PE (extra values within the interval 60–85% for juvenile mortality and within the interval 4–8% for adult mortality were included to highlight this relationship). The reported λ was calculated based on the stochastic growth rate (*stoc-r*).

Our results showed that decreases in juvenile mortality would favor a population increase, precluding the eagle population from extinction (λ>1) (Fig. 1a). Values of juvenile mortality of below 80% would avoid population extinction in the long term, taking into account values obtained either by satellite-tracking reported from Spanish populations [17] or by CMR methods from France [45] (Fig. 1b). If the values of juvenile mortality in Sicily were similar to those found in the Spanish and French populations, the Bonelli’s eagle population would become extinct in Italy within the next 100 years. Despite being close to the brink of extinction, the results of the sensitivity modelling reveal that any improvement aimed at decreasing juvenile mortality would allow population maintenance.

Similarly, the values of adult mortality determine the population’s fate (Fig. 1c). In our case, the current value of adult mortality recorded in Sicily was 10.2% [31], which is quite similar to the mortality rate obtained for France (13.0%). Therefore, it is not surprising that the results of the sensitivity analysis were roughly equal, regardless of the method used to estimate adult mortality. Only values of adult mortality below 3.0% (using data from Spain) or 4.5% (from France) would prevent population extinction (i.e., λ ≥1) (Fig. 1c). However, similar to juvenile mortality (Fig. 1b), the relationship between the probability of extinction (PE) and adult mortality does not follow a linear relationship but rather is sinusoidal (Fig. 1d). Therefore, very small changes in juvenile or adult mortality determine the population trend in the long term. Again, as with juvenile mortality, any improvements focused on decreasing adult mortality would prevent population extinction. Interestingly, the sensitivity analysis showed that a 30% decrease in juvenile mortality would increase the population growth rate by +7.79% (+6.82% using data from France), whereas a similar decrease in adult mortality would only raise the population growth rate by +2.53% (+3.15% using data from France) (Table 2). Hence, in comparative terms, the effectiveness of changing one parameter over the other would result in as much as a three-fold difference in the final population size. Logically, the highest effects on population growth rate would be obtained if both parameters were modified together. Notably, a hypothetical reduction of both adult and juvenile mortality by 30% would increase the population growth rate to +11.29%, using data from Spain, or to +10.85%, using data from France.

**Table 2:** Sensitivity values evaluated by the change in the population growth rate (λ) resulting from a given change in demographic parameters.

		Juvenile mortality		Adult mortality		Juvenile + Adult mortality		Breeding success	
Change		Spain	France	Spain	France	Spain	France	Spain	France
Increase	+30%	−5.07%	−4.50%	−2.57%	−2.86%	−6.95%	−7.32%	1.21%	1.61%
	+25%	−4.69%	−3.92%	−2.27%	−2.76%	−5.92%	−6.39%	1.11%	1.31%
	+20%	−3.82%	−3.25%	−1.78%	−2.08%	−5.07%	−5.26%	0.90%	1.21%
	+15%	−2.66%	−2.47%	−1.29%	−0.60%	−3.54%	−3.73%	0.80%	0.70%
	+10%	−1.88%	−1.78%	−0.80%	−1.00%	−2.57%	−2.66%	0.50%	0.50%
	+5%	−1.09%	−1.09%	−0.40%	−0.40%	−0.90%	−1.49%	0.10%	0.30%
Status quo	0	0.00%	0.00%	0.00%	0.00%	0.00%	0.00%	0.00%	0.00%
Decrease	−5%	1.01%	0.70%	0.40%	0.50%	2.02%	1.41%	−0.20%	−0.50%
	−10%	2.02%	1.71%	0.80%	1.11%	3.25%	2.84%	−0.70%	−0.60%
	−15%	3.46%	2.84%	1.26%	1.61%	5.87%	4.39%	−0.80%	−0.90%
	−20%	4.60%	3.87%	1.71%	2.12%	7.57%	6.61%	−1.19%	−1.39%
	−25%	6.18%	5.23%	2.12%	2.63%	9.97%	9.09%	−1.59%	−1.59%
	−30%	7.79%	6.82%	2.53%	3.15%	11.29%	10.85%	− 1.98%	−2.08%

Regarding breeding performance, Vortex simulations showed that variation in breeding success would also change population trends (Fig. 1e). The current value of breeding success was set at 60.15±21.05% (mean ± SD) according to intensive field sampling of the Sicilian population [31]. In contrast to the results found taking into account either adult or juvenile mortality, changes in the percentage of successful breeding pairs would not have the same influence in determining the population’s fate. Even when the parameter for breeding success was set at the maximum theoretical value of 100% (i.e., all pairs breeding successfully each year, which is quite unfeasible), the λ>1 threshold for population maintenance would not be reached (λ_Spain_ = 0.968; λ_France_ = 0.959) (Fig. 1e). Only the values of breeding success above 70% would allow the population to persist in the short term but with a high probability of extinction (PE>90%) (Fig. 1f). Furthermore, a 30% increase in breeding performance would only increase the population growth rate by +1.21% (+1.61% using data from France; Table 2).

Vortex simulations showed that deviations in the sex ratio only minimally changed population trends. As the sex ratio was deviated toward males, the population growth rate decreased slightly (Fig. 1g). Interestingly, the highest median time to extinction was obtained when the sex ratio deviated toward females (60% females:40% males) (median time of extinction *_Spain_* = 45 years; median time of extinction *_France_* = 39 years; *N* = 500 simulations). Nevertheless, changes in the sex ratio alone were not powerful enough to avoid population extinction if the current conditions persist (PE = 1 in all cases; Fig. 1h).

### Elasticity Analysis

**Figure 2:**
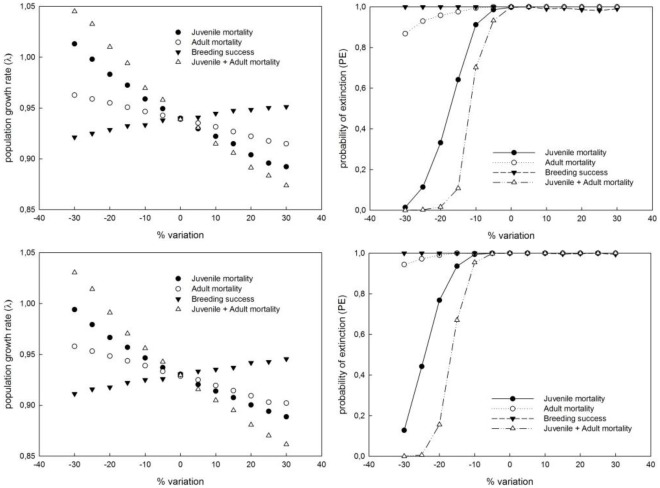
Elasticity analysis showing the variation in the population growth rate (λ) resulting from proportional changes in juvenile mortality, adult mortality, juvenile and adult mortality considered together, and breeding success. Simulations were performed using data from Spain (upper panel) and France (lower panel).

The comparative results of the elasticity analyses showed that similar changes in demographic parameters in relative terms had different results in determining the fate of the population (Fig. 2; Table S1). Our results showed that changes aimed either at decreasing adult mortality or at increasing breeding success had positive effects on demographic trends, allowing a relative population increase. For example, a 30% reduction in adult mortality would result in a decrease in PE from 100% to 86.8%, considering the Spanish baseline, or from 100% to 94.4%, considering the French baseline. While a 30% increase in breeding success would result in a change in PE from 100% to 99.0% (Spanish baseline) or from 100% to 99.4% (French baseline), the change would not be enough to prevent extinction (Fig. 2). However, only measures aimed at decreasing juvenile mortality alone or juvenile and adult mortality together would have a net positive effect on population persistence (i.e., λ>1; Fig. 2). A 30% decrease in juvenile mortality resulted in a PE = 1.4% in 100 years (λ *_Spain_* = 1.013) given the Spanish baseline or a PE = 12.8% given the French baseline (λ *_France_* = 0.994). Comparatively, the effectiveness of reducing juvenile mortality by 30% was 3.1 times more efficient than reducing adult mortality alone (*S_adult mortality_* = 2.53% vs. *S_juvenile mortality_* = 7.79%) given the Spanish baseline and 2.2 times (*S_adult mortality_* = 3.15% vs. *S_juvenile mortality_* = 6.82%) given the French baseline.

As expected, higher positive effects on population growth rate and PE were obtained when juvenile and adult mortality rates were decreased simultaneously. For example, a 20% decrease of both mortality rates resulted in positive population growth (λ *_Spain_* = 1.010; λ *_France_* = 1.014) and very low PE values (PE_Spain_ = 1.6%; PE_France_ = 0.6%) (Fig. 2; Table S1).

## Discussion

Recent research has revealed the extensive cascading effects caused by the disappearance of large top predators in terrestrial, marine and freshwater ecosystems worldwide, with far-reaching effects on ecological processes [50]. Top predators have been largely associated with high biodiversity areas [51] and have even been proposed as adequate surrogates for conservation [52]. In the Mediterranean region, a biodiversity hotspot, the Bonelli’s eagle can be considered an indicator of ecosystem health. The distribution of Bonelli’s eagles in the Mediterranean region has been associated with well-preserved habitats and, particularly, with the presence of healthy populations of key prey species, such as common rabbits *Oryctolagus cuniculus*, pigeons *Columba spp*., red partridges *Alectoris rufa* and lizards [53]–[56]. These prey species are, in turn, associated with the use of traditional agricultural and farming practices in the area [36], which are further key determinants of major biodiversity values found in the Mediterranean region [57]. Therefore, the disappearance of large top predators such as Bonelli’s eagle could give rise to detrimental effects not only for the species itself but also for the entire ecosystem.

In this paper, we showed the results of an extinction risk assessment for an isolated, small population of a large top predator species in southern Europe. While most studies on demography and population dynamics of Bonelli’s eagle have been occurred in Spain [e.g., 17–19], recently, demographic studies have been extended to the population of eagles in southern France [45], [46]. There is a lack of information about other regions across the eagle’s distribution range and this is the first attempt to specifically estimate the extinction risk of Bonelli’s eagles by a comprehensive PVA in Italy. In summary, our results highlight that measures aimed at decreasing juvenile and adult mortality rather than those focused on improving breeding success are needed to ensure the long-term persistence of the species. These findings have important consequences not only for the conservation of this species in particular but also for the conservation of endangered species in general. Our approach can also serve as a model of extinction risk assessment for large top predators.

### Uncertainty in Parameter Estimation

PVAs play a key role in the conservation management decision-making process, even under scenarios where there is great uncertainty [58], [59]. A consistent body of literature suggests that PVAs can be used to quantify extinction risks [5], [6], [60]. The estimation of the risk of extinction is calculated based on estimates of demographic parameters, which are usually calculated in probabilistic terms. Therefore, there is an inherent uncertainty in the construction of PVAs [11], [61] and care should be taken in their interpretation [49], [62]. Yet, coping with uncertainty is intrinsically linked to the activity of conservation biologists, who ought to give advice to managers which, in theory, need taking decisions based on unequivocal, evidence-based scientific prescriptions [63].

Models are simplifications of reality; consequently, their output should be interpreted cautiously [4]. That is, our estimations of population extinction and growth rates should be considered cautiously given that they are only projections rather than real values based on deterministic estimations of the input parameters. This is essential when translating the results of PVAs to conservation practitioners, who can be prone to interpret simulation data as real predictions [11]. One of the main shortcomings of Vortex is that it does not provide the capacity for inputting parametric uncertainty into model projections [58]. Therefore, our results should be interpreted to show projections of population trends according to variations in demographic parameters, and thus, it is beyond our scope to provide accurate predictions of when populations will become extinct and/or estimate the minimum viable population size required for long-term persistence [4]. In contrast, one of the main advantages of Vortex is the inclusion of stochastic variations in demographic parameters so that several sources of annual variation are intrinsically considered in simulations [40]. Although it is possible that temporal variations in some parameters could arise a posteriori (they likely will), this does not invalidate the use of PVAs to assess the extinction risk of endangered species [6], [64].

### Conservation Implications

Obtaining robust estimates of demographic parameters is essential to gain insight into the demographic dynamics of endangered species [65]. There is a general agreement that survival, rather than breeding performance, is the major determinant of the persistence of populations of large predators [66], particularly for Bonelli’s eagle [17], [19], [45]. This is typical of long-lived birds, with deferred sexual maturity and low clutch size [67]. The main limitation of our population models was the uncertain accuracy of survival rate estimates. Adult mortality rates in Sicily (10.2%) were similar to several Bonelli’s eagle populations in the Iberian peninsula (3% – 16%; [17], [68]) and France (12% – 13% [45], [46]). Therefore, even when taking into account geographical variation in vital rates, our results are consistent demographically. Unfortunately, specific juvenile survival rates for the Sicilian population of Bonelli’s eagle are not currently available. To overcome this limitation, we used data obtained through different methods, such as the satellite-tracking program of juvenile birds in eastern Spain [17], [44] and juvenile and adult mortality values obtained by systematic CMR methods in southern France [45]. Interestingly, our results showed similar decreasing population trends for the Sicilian population, regardless of the source of data used for modelling. The sinusoidal shape of the relationship between the probability of extinction and the range of values for adult mortality and juvenile mortality is remarkable (Fig. 1). This sinusoidal relationship indicates that when the rest of the parameters remain unchanged (i.e., if current conditions are to persist), only the values of juvenile mortality that are below 80% would avoid extinction in the next 100 years. From a management perspective, it should be emphasised that small changes in juvenile mortality (especially those included in the interval 70% – 80%) or in adult mortality (those ranging from 4% to 9%) notably change the population’s fate. This point is crucial from a conservation point of view because it provides essential information to optimise the decision-making process, indicating that measures aimed at decreasing juvenile and adult mortality, either separately or jointly, are urgently needed to ensure the long-term persistence of the species in Italy. The elasticity analysis showed that juvenile mortality alone is the most critical demographic parameter, with the strongest influence on population persistence (Fig. 2), when compared to the relative changes in population trends obtained when management actions were aimed at either decreasing both juvenile and adult mortality, or increasing breeding success (Table 2).

Our population model results for Bonelli’s eagle in Italy stress the emergent key role of juvenile mortality on population persistence [17]. This point has important implications for management and conservation for this critically endangered species on a broader scale. In fact, our results could be generalised to the entire range of Bonelli’s eagle, thus becoming a focal point for concrete actions in a new European Action Plan for the species as well as a basis for the management of other small populations of long-lived species.

Currently, Bonelli’s eagle faces several conservation problems across its distribution range, mainly due to habitat loss and habitat transformation, which are particularly significant in Italy [31]. Other than habitat loss, direct persecution from poaching (a minimum of 14 cases in the last 20 years, authors unpub. data) and nest plundering for falconry and collection (16 cases compiled in the last 4 years, authors unpub. data) constitute the main causes of mortality of adult and juvenile Bonelli’s eagles in our study area. This adds to other mortality causes such as a loss of prey species, increased human pressure on breeding habitats and even poisoning. The combined effects of all threats are impacting the last breeding pairs of the species on the island of Sicily. Therefore, urgent tasks, such as the removal of sources of direct persecution, particularly poaching and nest plundering via legal punishment and increased control by the forestry police authorities, are essential to guarantee the viability of the population in Italy. In addition, other measures aimed at reducing juvenile mortality, such as the correction of dangerous electric pylons, have been demonstrated to be highly efficacious in overcoming declining population trends of other endangered raptors such as the Spanish Imperial eagle (*Aquila adalberti*) [66], [69]. Finally, the cessation of habitat transformations and the increase of prey availability through the promotion of traditional land use and sensible game management should also be encouraged [32], [70]–[72]. At present, Bonelli’s eagle, despite its national and European conservation concern [21], lacks a specific conservation plan in Italy, and the European Action Plan [73] requires updating and implementation. Therefore, we recommend the urgent onset of a specific broad-scale conservation program including intensive research into the species’ geographic distribution. Finally, both proactive and reactive management actions focused on reducing the mortality causes should be undertaken.

## Supporting Information

Table S1 **Elasticity analysis resulting from proportional changes in juvenile mortality, adult mortality, both juvenile and adult mortality and breeding success.** Two different baseline models were considered: one using juvenile mortality recorded in eastern Spain [15] and adult mortality from the Sicilian population [31]; and other including juvenile and adult mortality from southern France [44]. The results of similar analyses are shown in adjacent columns to allow comparisons. Time of extinction expressed in years. (See text for further details). Positive values indicate that the change causes population to increase, whereas negative values indicate that the change causes population to decrease. Simulations were run under two different scenarios, considering mortality values either from Spain and Italy, or from France (See text for details).
(DOC)
