# Correction: Living on the Edge: Assessing the Extinction Risk of Critically Endangered Bonelli’s Eagle in Italy

**DOI:** 10.1371/annotation/dd99f4a8-9ec1-4177-9b2b-e145a348ca2a

**Published:** 2012-08-27

**Authors:** Pascual López-López, Maurizio Sarà, Massimiliano Di Vittorio

There was an error in affiliation 1 for author Pascual López-López. Affiliation 1 should be: Vertebrates Zoology Research Group, CIBIO, University of Alicante, Alicante, Spain 

