# Supplementary material for: Living on the Edge: Assessing the Extinction Risk of Critically Endangered Bonelli’s Eagle in Italy
Source: PLoS One. 2012 May 25;7(5):e37862. doi: 10.1371/journal.pone.0037862 (PMC3360590; doi:10.1371/journal.pone.0037862)
Supplement: Table S1 — Elasticity analysis resulting from proportional changes in juvenile mortality, adult mortality, both juvenile and adult mortality and breeding success. Two different baseline models were considered: one using juvenile mortality recorded in eastern Spain [15] and adult mortality from the Sicilian population [31]; and other including juvenile and adult mortality from southern France [44]. The results of similar analyses are shown in adjacent columns to allow comparisons. Time of extinction expressed in years. (See text for further details). Positive values indicate that the change causes population to increase, whereas negative values indicate that the change causes population to decrease. Simulations were run under two different scenarios, considering mortality values either from Spain and Italy, or from France (See text for details). (DOC) [file pone.0037862.s001.doc]

**Table S1.**

| **Scenario** | **Deterministic-r** | | **Stochastic-r (SD)** | | **Probability of extinction** | | **Median time of extinction** | | **Mean time of extinction** | |
| --- | --- | --- | --- | --- | --- | --- | --- | --- | --- | --- |
|  | Spain | France | Spain | France | Spain | France | Spain | France | Spain | France |
| Baseline model Spain | -0.059 | x | -0.064 (0.187) | x | 1.000 | x | 44 | x | 45.0 | x |
| Baseline model France | x | -0.067 | x | -0.074 (0.177) | x | 1.000 | x | 38 | x | 39.4 |
| Juvenile mortality +30% | -0.174 | -0.140 | -0.114 (0.202) | -0.118 (0.183) | 1.000 | 1.000 | 24 | 24 | 24.8 | 25.2 |
| Juvenile mortality +25% | -0.151 | -0.128 | -0.110 (0.205) | -0.112 (0.185) | 1.000 | 1.000 | 24 | 25 | 26.1 | 26.6 |
| Juvenile mortality +20% | -0.126 | -0.115 | -0.101 (0.206) | -0.105 (0.188) | 1.000 | 1.000 | 28 | 27 | 29.3 | 28.4 |
| Juvenile mortality +15% | -0.102 | -0.100 | -0.089 (0.199) | -0.097 (0.180) | 1.000 | 1.000 | 32 | 30 | 33.2 | 30.4 |
| Juvenile mortality +10% | -0.090 | -0.091 | -0.081 (0.194) | -0.090 (0.183) | 1.000 | 1.000 | 35 | 32 | 35.7 | 32.8 |
| Juvenile mortality +5% | -0.077 | -0.079 | -0.073 (0.189) | -0.083 (0.180) | 1.000 | 1.000 | 38 | 34 | 39.4 | 35.1 |
| Juvenile mortality -5% | -0.045 | -0.055 | -0.052 (0.179) | -0.065 (0.176) | 0.986 | 1.000 | 52 | 43 | 54.3 | 44.4 |
| Juvenile mortality -10% | -0.032 | -0.044 | -0.042 (0.171) | -0.055 (0.170) | 0.912 | 0.994 | 63 | 49 | 61.8 | 50.9 |
| Juvenile mortality -15% | -0.015 | -0.029 | -0.028 (0.155) | -0.044 (0.160) | 0.642 | 0.936 | 89 | 60 | 73.5 | 59.8 |
| Juvenile mortality -20% | -0.006 | -0.020 | -0.017 (0.140) | -0.034 (0.154) | 0.332 | 0.768 | - | 75 | 74.3 | 68.0 |
| Juvenile mortality -25% | 0.006 | -0.008 | -0.002 (0.121) | -0.021 (0.135) | 0.114 | 0.442 | - | - | 74.1 | 71.1 |
| Juvenile mortality -30% | 0.018 | 0.004 | 0.013 (0.107) | -0.006 (0.114) | 0.014 | 0.128 | - | - | 81.4 | 73.5 |
| Adult mortality +30% | -0.088 | -0.100 | -0.089 (0.196) | -0.103 (0.188) | 1.000 | 1.000 | 32 | 27 | 32.6 | 28.4 |
| Adult mortality +25% | -0.083 | -0.095 | -0.086 (0.197) | -0.102 (0.188) | 1.000 | 1.000 | 33 | 28 | 34.1 | 29.4 |
| Adult mortality +20% | -0.079 | -0.089 | -0.081 (0.194) | -0.095 (0.187) | 1.000 | 1.000 | 36 | 30 | 36.4 | 30.7 |
| Adult mortality +15% | -0.067 | -0.076 | -0.076 (0.192) | -0.080 (0.179) | 1.000 | 1.000 | 41 | 35 | 41.9 | 36.0 |
| Adult mortality +10% | -0.069 | -0.078 | -0.071 (0.192) | -0.084 (0.180) | 1.000 | 1.000 | 40 | 33 | 41.3 | 34.4 |
| Adult mortality +5% | -0.064 | -0.073 | -0.067 (0.189) | -0.078 (0.178) | 1.000 | 1.000 | 42 | 36 | 43.3 | 37.5 |
| Adult mortality -5% | -0.055 | -0.062 | -0.059 (0.183) | -0.069 (0.175) | 1.000 | 1.000 | 47 | 41 | 48.3 | 41.8 |
| Adult mortality -10% | -0.050 | -0.056 | -0.055 (0.179) | -0.063 (0.174) | 0.994 | 0.998 | 50 | 44 | 51.5 | 45.8 |
| Adult mortality -15% | -0.039 | -0.051 | -0.051 (0.169) | -0.058 (0.169) | 0.956 | 1.000 | 60 | 47 | 61.0 | 49.7 |
| Adult mortality -20% | -0.041 | -0.046 | -0.046 (0.178) | -0.053 (0.166) | 0.958 | 0.990 | 59 | 51 | 60.2 | 53.0 |
| Adult mortality -25% | -0.036 | -0.040 | -0.042 (0.170) | -0.048 (0.163) | 0.930 | 0.972 | 65 | 57 | 64.1 | 57.4 |
| Adult mortality -30% | -0.031 | -0.035 | -0.038 (0.167) | -0.043 (0.158) | 0.868 | 0.944 | 70 | 62 | 67.0 | 62.1 |
| Juvenile & Adult mortality +30% | -0.202 | -0.176 | -0.135 (0.197) | -0.149 (0.194) | 1.000 | 1.000 | 23 | 20 | 21.9 | 20.3 |
| Juvenile & Adult mortality +25% | -0.169 | -0.157 | -0.124 (0.201) | -0.139 (0.191) | 1.000 | 1.000 | 23 | 22 | 23.9 | 21.8 |
| Juvenile & Adult mortality +20% | -0.143 | -0.138 | -0.115 (0.198) | -0.127 (0.189) | 1.000 | 1.000 | 24 | 23 | 25.6 | 23.8 |
| Juvenile & Adult mortality +15% | -0.117 | -0.120 | -0.099 (0.201) | -0.111 (0.189) | 1.000 | 1.000 | 28 | 25 | 30.0 | 26.4 |
| Juvenile & Adult mortality +10% | -0.097 | -0.102 | -0.089 (0.199) | -0.100 (0.182) | 1.000 | 1.000 | 32 | 27 | 32.9 | 28.9 |
| Juvenile & Adult mortality +5% | -0.075 | -0.085 | -0.072 (0.190) | -0.088 (0.181) | 1.000 | 1.000 | 39 | 32 | 40.2 | 33 |
| Juvenile & Adult mortality -5% | -0.034 | -0.050 | -0.043 (0.171) | -0.059 (0.171) | 0.932 | 0.998 | 63 | 47 | 61.7 | 48 |
| Juvenile & Adult mortality -10% | -0.021 | -0.033 | -0.031 (0.160) | -0.045 (0.162) | 0.702 | 0.954 | 82 | 61 | 70.5 | 60.5 |
| Juvenile & Adult mortality -15% | 0.001 | -0.016 | -0.006 (0.122) | -0.030 (0.145) | 0.108 | 0.670 | - | 85 | 80.7 | 71.6 |
| Juvenile & Adult mortality -20% | 0.013 | 0.000 | 0.010 (0.105) | -0.009 (0.115) | 0.016 | 0.156 | - | - | 84.9 | 79.5 |
| Juvenile & Adult mortality -25% | 0.034 | 0.017 | 0.032 (0.096) | 0.014 (0.090) | 0.002 | 0.006 | - | - | 77.0 | 77.3 |
| Juvenile & Adult mortality -30% | 0.045 | 0.033 | 0.044 (0.094) | 0.030 (0.084) | 0.000 | 0.000 | - | - | - | - |
| Breeding success +30% | -0.037 | -0.042 | -0.050 (0.173) | -0.056 (0.163) | 0.990 | 0.994 | 53 | 48 | 55.5 | 49.9 |
| Breeding success +25% | -0.040 | -0.046 | -0.051 (0.176) | -0.059 (0.164) | 0.982 | 1.000 | 53 | 46 | 54.6 | 47.4 |
| Breeding success +20% | -0.044 | -0.050 | -0.053 (0.178) | -0.060 (0.161) | 0.986 | 0.998 | 52 | 45 | 52.2 | 46.1 |
| Breeding success +15% | -0.046 | -0.054 | -0.054 (0.177) | -0.065 (0.172) | 0.994 | 0.996 | 50 | 42 | 51.7 | 43.7 |
| Breeding success +10% | -0.051 | -0.058 | -0.057 (0.182) | -0.067 (0.167) | 0.990 | 1.000 | 49 | 41 | 50.0 | 42.8 |
| Breeding success +5% | -0.055 | -0.063 | -0.061 (0.182) | -0.069 (0.170) | 1.000 | 1.000 | 45 | 40 | 47.0 | 41.3 |
| Breeding success -5% | -0.064 | -0.072 | -0.064 (0.184) | -0.077 (0.171) | 1.000 | 1.000 | 44 | 37 | 45.2 | 38.0 |
| Breeding success -10% | -0.068 | -0.077 | -0.069 (0.185) | -0.078 (0.170) | 1.000 | 1.000 | 41 | 36 | 42.7 | 37.2 |
| Breeding success -15% | -0.071 | -0.082 | -0.070 (0.188) | -0.081 (0.179) | 1.000 | 1.000 | 41 | 34 | 41.7 | 35.7 |
| Breeding success -20% | -0.078 | -0.087 | -0.074 (0.190) | -0.086 (0.171) | 1.000 | 1.000 | 38 | 33 | 39.0 | 34.3 |
| Breeding success -25% | -0.083 | -0.092 | -0.078 (0.190) | -0.088 (0.171) | 1.000 | 1.000 | 37 | 33 | 38.1 | 33.6 |
| Breeding success -30% | -0.088 | -0.098 | -0.082 (0.190) | -0.093 (0.170) | 1.000 | 1.000 | 35 | 30 | 36.2 | 31.6 |
